# Retraction: Natural killer cell-derived exosomal miR-3607-3p inhibits pancreatic cancer progression by targeting IL-26

**DOI:** 10.3389/fimmu.2024.1480124

**Published:** 2024-08-19

**Authors:** Frontiers Editorial Office

Following publication, concerns were raised regarding the integrity of the images in the published figures. The authors failed to provide a satisfactory explanation during the investigation, which was conducted in accordance with Frontiers’ policies.

This retraction was approved by the Chief Editors of Frontiers in Immunology and the Chief Executive Editor of Frontiers. The authors agree to this retraction.

